# Successful induction of diabetes in mice demonstrates no gender difference in development of early diabetic retinopathy

**DOI:** 10.1371/journal.pone.0238727

**Published:** 2020-09-17

**Authors:** Aicha Saadane, Emma M. Lessieur, Yunpeng Du, Haitao Liu, Timothy S. Kern

**Affiliations:** 1 Department of Ophthalmology, University of California-Irvine, Irvine, California, United States of America; 2 Department of Ophthalmology, Children's Hospital of University of Pittsburgh School of Medicine, Pittsburgh, Pennsylvania, United States of America; 3 Veterans Administration Medical Center Research Service, Long Beach, California, United States of America; University of Florida, UNITED STATES

## Abstract

**Purpose:**

Female mice have been found to be resistant to streptozotocin (STZ)-induced diabetes, and pre-clinical research related to diabetic complications commonly omits females. The purpose of this study was to develop a method to induce diabetes in female mice, and to determine if retinas of diabetic female mice develop molecular changes and histopathological abnormalities comparable to those which develop in male diabetic mice.

**Methods:**

To induce diabetes, animals of both sexes received daily intraperitoneal (i.p.) injection of STZ for 5 consecutive days at 55 mg/kg BW (a dose that is known to induce diabetes in male mice) or for females, 75 mg/kg BW of STZ. Retinal abnormalities that have been implicated in the development of the retinopathy (superoxide generation and expression of inflammatory proteins, iNOS and ICAM-1) were evaluated at 2 months of diabetes, and retinal capillary degeneration was evaluated at 8 months of diabetes.

**Results:**

Daily i.p. injection of STZ for 5 consecutive days at a concentration of 55 mg/kg BW was sufficient to induce diabetes in 100% of male mice, but only 33% of female mice. However, females did become hyperglycemic when the dose of STZ administered was increased to 75 mg/kg BW. The resulting STZ-induced hyperglycemia in female and male mice was sustained for at least 8 months. After induction of the diabetes, both sexes responded similarly with respect to the oxidative stress, expression of iNOS, and degeneration of retinal capillaries, but differed in the limited population evaluated with respect to expression of ICAM-1.

**Conclusions:**

The resistance of female mice to STZ-induced diabetes can be overcome by increasing the dose of STZ used. Female mice can, and should, be included in pre-clinical studies of diabetes and its complications.

## Introduction

Differences between males and females clearly do exist with regard to sensitivity or response to drugs and diseases [1], making it critical to study both sexes in pre-clinical studies. Accordingly, NIH now stipulates that investigators should use both sexes in their research.

Although diabetes is known to affect both sexes in patients and in some genetic mouse models, many basic science studies of diabetic complications have focused on STZ-induced diabetes using only male mice. Female mice have been largely omitted from this research, at least in part, because they have been reported to be resistant to the diabetogenic actions of chemicals like streptozotocin [2–6]. Moreover, the possibility that hormone fluctuations during the reproductive cycle could add undesirable variability to experimental studies further lessened efforts to use female rodents in studies of diabetic complications.

In this study, we confirm that female C57Bl/6J mice are significantly more resistant to diabetogenic effects of STZ than male counterparts, and such resistance can be overcome merely by increasing the amount of STZ administered to females. The resulting diabetes can be maintained for at least 8 months, and the diabetes-induced molecular abnormalities in retina and leukocytes, and the subsequent degeneration of retinal capillaries, are similar in both sexes.

## Materials and methods

### Mice

Male and female wild-type (WT; C57BL/6J) mice were obtained from the Jackson Laboratory. This study was performed in strict accordance with the National Institutes of Health Guide for the Care and Use of Laboratory Animals, the Association for Research in Vision and Ophthalmology Statement for the Use of Animals in Ophthalmic and Vision Research, and with authorization of Case Western Reserve University-, and University of California Irvine Institutional Animal and Care Use Committees (IACUC). All efforts were made to minimize suffering within the context of the diabetic protocol including administration of insulin to prevent weight loss.

### Induction of diabetes

Diabetes was induced between 10 and 12 weeks of age in males and between 10 and 12 weeks in females by intraperitoneal injection (i.p.) of a freshly prepared solution of streptozotocin (STZ) in citrate buffer (55 mg/kg body weight [BW] for 5 consecutive days). Other female mice were subjected to i.p. injection of STZ for 5 consecutive days at a dose of either 55 or 75 mg/kg BW. Hyperglycemia was verified at least three times during the second week after streptozotocin administration, and mice having three consecutive measurements of blood glucose >275 mg/dL were classified as being diabetic.

Insulin (0–0.2 units of NPH insulin s.c., 0–3 times per week) was given to diabetic animals as needed to prevent weight loss without hampering hyperglycemia or glucosuria. All animals were maintained on a standard 12-h light-dark cycle and were provided standard rodent chow (Purina TestDiet 5001; Richmond, IN) and water ad libitum. Blood glucose and HbA1c (hemoglobin A1c) were measured as reported previously [7, 8]. Blood was drawn from the left ventricle into a capillary tube and stored in an Eppendorf tube with a small amount of heparin to prevent coagulation.

#### Retinal imaging and visual function

Spectral-domain optical coherence tomography (SD-OCT; Bioptigen, Durham, NC) was used for *in vivo* imaging of mouse retinas, as reported previously [9]. Briefly, mice were anesthetized by i.p. injection of ketamine/xylazine (10 mg/100 g BW/1 mg/100 g BW). Pupils were dilated with 1% tropicamide. Five pictures acquired in the B-scan mode were used to construct each final averaged image. Thickness of the outer nuclear layer (ONL) was measured at distances of 0.15, 0.30, and 0.45 mm from the optic nerve, and the thickness of nerve fiber layer (NFL), inner plexiform layer (IPL), inner nuclear layer (INL), and total retinal were measured at distance of 0.30 mm from the optic nerve. The average thickness from the disc is reported. Electroretinographic (ERG, Diagnosys Celeris rodent ERG device, Diagnosys, Lowell, MA) recordings were performed as described [10], except that mice were anesthetized using isoflurane.

### Lucigenin assay of superoxide

Superoxide levels were measured chemically with lucigenin (bis-*N*-methylacridinium nitrate), as reported previously [11]. Briefly, freshly isolated retinas were incubated in 200 ml of Krebs-Hepes buffer (pH 7.2) with 5 or 30 mM glucose for 7 min in 37°C in 5% CO2. Luminescence indicating the presence of superoxide was measured 7 min after addition of lucigenin. Luminescence intensity is reported in arbitrary units per mg of protein.

### Retina explants

Eyes were enucleated from adult nondiabetic C57Bl/6J mice and immediately immersed in ice-cold PBS containing 10% FBS, 100 units/ml penicillin, and 100 μg/ml streptomycin. Retinas were isolated and incubated for 24 hours in Neurobasal media (Invitrogen, catalogue# A244775, Waltham, MA) supplemented with B27 (Invitrogen, catalogue# 10889038, Waltham, MA), 0.5 mM L-glutamine, 100 units/ml penicillin/100 μg/ml streptomycin, and either 5 mM or 25 mM D-glucose (Sigma, catalogue# G8769, St. Louis, MO) or D-mannitol (osmotic control) (Sigma, catalogue# SLCD7105, St. Louis, MO) in a humidified incubator with 5% CO_2_ at 37°C. At the end of this incubation, the retina was used to assay for superoxide levels as described above.

### Western blotting

One retina from each mouse was sonicated in 70 μl of lysis buffer (50mM Tris, pH 8.0, 150 mM NaCl, 5 mM EDTA, 1% Nonidet P-40, 0.1% SDS, and complete EDTA-free protease inhibitor mixture from Roche, Indianapolis, IN). Retinal homogenates were incubated on ice for 30 min followed by centrifugation at 12,000 x g for 15 min at 4°C. The supernatant was used for SDS-PAGE (50 μg protein/lane) followed by western blotting, which was performed as described [12]. Proteins were visualized with the following primary antibodies: 1:200 for Adhesion Molecule-1 (ICAM-1) (Santa Cruz Biotechnology Catalogue# sc-71292, Santa Cruz, CA), 1:200 for iNOS (BD Biosciences Catalogue# 610328, San Jose, CA). The secondary antibody was goat anti-rabbit IRDye 800CW (925–32211, Li-Cor, dilution 1:5,000). Membranes were also incubated with primary antibody against β-actin (1:5000), which was used as a loading control (Abcam Catalogue# ab8226, Cambridge, MA), and secondary goat anti-mouse IRDye 680RD (925–68070, Li-Cor, dilution 1:5000). Membranes were imaged using the Odyssey infrared imaging system (Li-Cor, Lincoln, NE).

### Diabetes-induced retinal histopathology

After 8 months of diabetes, mouse eyes were fixed in formalin, and one retina from each animal was isolated, washed in running water overnight, and digested for at least two and half hours in elastase as we previously reported [13]. When totally cleaned of neural cells, the isolated vasculature was laid out on a glass microscope slide, dried overnight, stained with hematoxylin and periodic acid-Schiff, dehydrated, and mounted with a glass coverslip. Degenerated (acellular) capillaries were quantitated in 6–7 field areas corresponding to the mid-retina (200 x magnification) in a masked manner. Acellular capillaries reported per square millimeter of retinal area were identified as capillary-sized vessel tubes having no nuclei along their length.

### Leukocyte-mediated cytotoxicity toward endothelial cells

Transformed mouse retinal endothelial cells [14] were grown in control medium (Dulbecco's modified Eagle medium (DMEM) with 5 mM glucose) containing 10% serum. The serum concentration was reduced to 2% just before cells were placed either in 5 mM glucose or high glucose (30 mM). Media was changed every other day for 3 days. When cells reached 80% confluency (∼300,000 cells), freshly isolated leukocytes from blood (100,000 cells) were added and incubated for 6 additional hours, after which cells were collected and washed with PBS. Cells were labeled with an antibody against CD144 (1:50 dilution; BD Biosciences Pharmingen, San Diego, CA) to identify endothelial cells, and the viability of the endothelial cells was identified by flow cytometry based on 7-AAD staining. Endothelial cell death was expressed as the percentage of endothelial cells that stained positively with dye. Approximately 10,000 events were counted for each sample. Results were analysed using Flow Jo v7.6 Software (FlowJo, Ashland, OR).

### Statistical analysis

All statistical analyses were performed with analysis of variance (ANOVA) followed by Fisher’s test (StatView for Windows, SAS Institute Inc.) except for retinal explant, which was analyzed by a non-parametric Kruskal-Wallis test followed by Man-Whitney test, and for ERG, which was analyzed by two-way repeated-measures of variance. Data are expressed as mean ± SD.

## Results

### Female C57Bl/6J mice are significantly more resistant to diabetogenic effects of streptozotocin than male counterparts

To induce diabetes, male and female mice received daily intraperitoneal injection of 55 mg/kg BW for 5 consecutive days. As well documented in the literature, male mice responded to this dose of STZ with severe hyperglycemia, whereas female mice were more resistant to streptozotocin and most failed to develop diabetes (Table 1).

**Table 1:** Glycemia in male and female nondiabetic and diabetic mice 8 weeks post i.p. infections of 55 mg/kg body weight (mean ± SD).

Group	n	Final BW (g)	Fasting blood glucose, (mg/dl)	Mice that became diabetic
Male				
Nondiabetic	5	35 ± 1.1	194 ± 21.9	
Diabetic	7	27 ± 1.8	547 ± 65.9	7 out of 7
Female				
Nondiabetic	9	22 ± 0.8	130 ± 21.0	
Diabetic	12	21 ± 1.3	237 ± 86.3	3 out of 12

### Administration of a higher dose of streptozotocin to female mice resulted in meaningful hyperglycemia that could be maintained for at least 8 months

**Fig 1:**
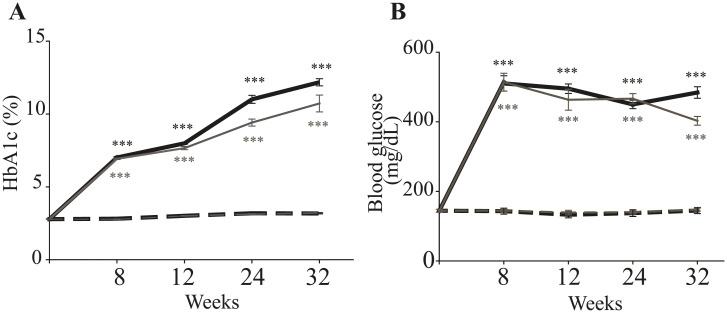
Glycemia following administration of STZ to female mice at a dose of 75 mg/kg BW (grey lines) compared to males at 55 mg/kg BW (bold black lines). In both sexes, the increase in (A) HbA1c or (B) blood glucose persisted for at least 8 months. Solid lines represent diabetic mice, and dashed lines represent nondiabetic control mice. Data are presented as mean ± SD (n = 8–13 per group). *** P ≤ 0.001.

To overcome the apparent resistance of female mice to the diabetogenic effects of STZ, another group of females were injected with a higher dose of the drug (75 mg/kg BW) for 5 consecutive days. This dose of streptozotocin resulted in a significant increase in blood glucose and in HbA1c in the female mice versus their nondiabetic controls. To investigate whether the STZ -induced hyperglycemia in these female mice persisted, we conducted a longitudinal study for 32 weeks after the mice were declared diabetic. Female mice injected with the higher dose of STZ showed a significant increase in fasting blood glucose and HbA1c by 8 weeks after the course of STZ, which continued to increase over the ensuing months (Fig 1A and 1B). The severity of hyperglycemia was similar between the 2 sexes, but blood sugar was significantly lower in females than in males at 32 weeks of diabetes (P = 0.002).

### Diabetic male and female mice developed similar abnormalities in retinal oxidative stress and expression of pro-inflammatory protein iNOS

**Fig 2:**
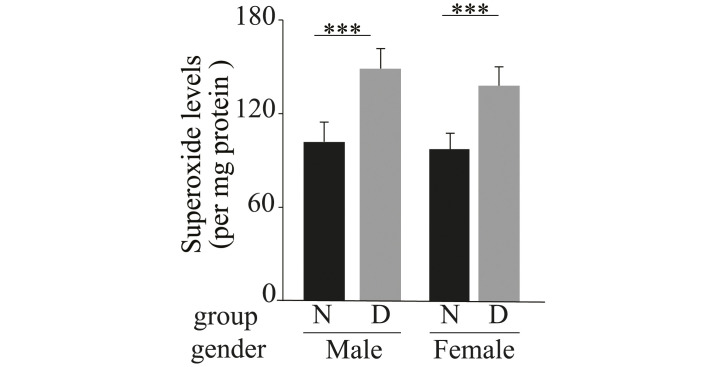
Two month duration of diabetes increased superoxide levels in retinas of male and female mice compared to their respective nondiabetic controls. Data are presented as mean ± SD (n = 5–6 per group). *** P ≤ 0.001. N; nondiabetic, D; diabetic.

Having demonstrated that female mice can become satisfactorily diabetic after STZ, next we sought to determine if retinas from female mice respond to the hyperglycemia in a manner comparable to that seen in male diabetics. Diabetes has been reported to result in a significant increase in retinal oxidative stress and induction of pro-inflammatory proteins in male mice [15–19], and both abnormalities have been implicated in the development of the retinopathy [17, 20, 21]. Thus, we focused on these two abnormalities. As expected, diabetes of 2 months duration increased the superoxide in retinas from male diabetics compared to their nondiabetic controls (148 ± 8.5 (arb units) versus 100 ± 9.3 in diabetic and nondiabetic males respectively) (Fig 2). Likewise, diabetes of 2 months duration also increased the superoxide in female retinas compared to their nondiabetic controls (138 ± 9.6 (arb units) versus 100 ± 10.1 in diabetic and nondiabetic females respectively) (Fig 2). This increase in retinal superoxide required chronic diabetes, because it was not reproduced by acute incubation of retinas from diabetic animals in 25mM glucose (100 ± 14.92 arb units versus 118 ± 16.10 arb units in 25mM and 5mM respectively, P = 0.318).

**Fig 3:**
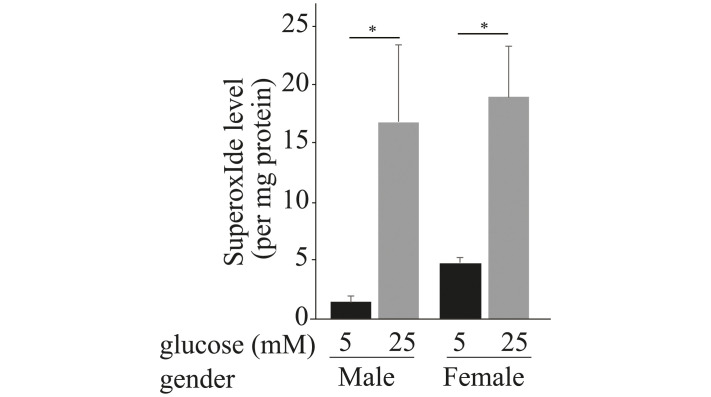
Retinal explants from nondiabetic female and male mice incubated in 5 or 25 mM glucose for 24 hours reveal that retinal superoxide in high glucose is elevated similarly in both sexes. Eyecups were obtained from wild type male and female mice at age 2–3 months. Data are presented as mean ± SD, n = 6–9 per group. * P ≤ 0.05.

To evaluate hyperglycemia-induced retinal generation of superoxide in the absence of other factors that might differ between the sexes, retinal explants from nondiabetic male and female mice were incubated in Neurobasal media containing diabetic-like (25mM) or nondiabetic (5mM) concentrations of glucose for 24 hours. A different set of retinas were incubated with D-mannitol (osmotic control). Assay of superoxide then showed that retinas from female mice produced a comparable increase in superoxide to that observed in retinas from male mice (Fig 3). The retinas incubated in 25 mM D-mannitol were comparable to control. These data suggest that the capacity of the retina to generate oxidative stress in elevated glucose is not influenced appreciably by sex-dependent programing in the retina.

**Fig 4:**
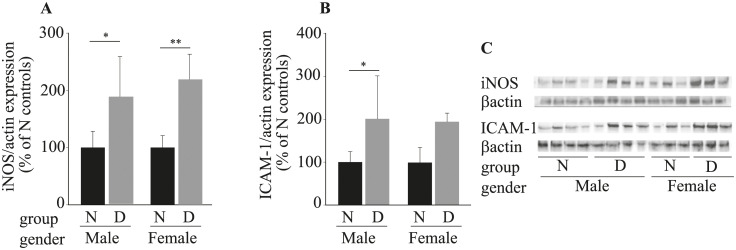
Effects of STZ-induced diabetes on protein expression of iNOS and ICAM-1 in retinas of female and male mice diabetic for 8 months. Summary graph for iNOS (A) and ICAM-1 (B) expression determined by image analysis. C representative immunoblots for iNOS, ICAM-1 and βactin. Data are expressed relative to the expression of βactin, a housekeeping protein in the same sample, and are expressed as a percent of the value of nondiabetic controls. Data are expressed as mean ± SD (n = 3–4). N, nondiabetic; D, diabetic. * P ≤ 0.05, ** P ≤ 0.001.

Since increased expression of proinflammatory proteins also has been implicated in the pathogenesis of the retinopathy in studies of male mice [21, 22], we compared the effects of diabetes on the expression of inflammatory proteins iNOS and ICAM-1 in diabetic male and female mice. As previously reported, diabetes of 2 months duration resulted in a significant increase in expression of retinal iNOS and ICAM-1 in diabetic male mice. Likewise, diabetes of 2 months duration resulted in a significant increase in expression of retinal iNOS in diabetic female mice (Fig 4A). Expression of ICAM-1 tended to show a similar pattern (Fig 4B), but the result was not statistically significant in the females.

### 8 months duration of diabetes did not affect retinal structure in either male or female mice

**Fig 5:**
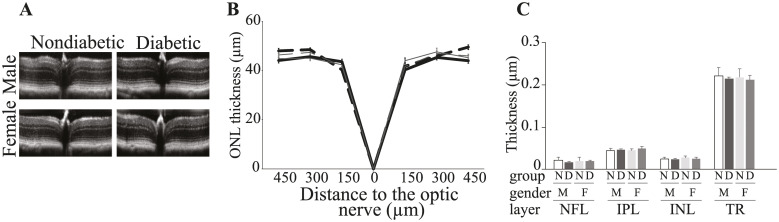
SD-OCT images (A) and quantification of data (B and C) show that 8 months duration of diabetes (dashed lines) resulted in essentially no loss of retinal photoreceptors (ONL thickness) in either male (bold black) or female (grey) mice compared to gender- and age-matched nondiabetic controls (solid lines). Similarly, TRL, NFL, IPL, and INL were not significantly different between diabetic and nondiabetic. Data are presented as mean ± SD, n = 4 mice (8 retinas) per group. N, nondiabetic; D, diabetic; M, males; F, females; TR, total retina; NFL, nerve fiber layer; IPL, inner plexiform layer; INL, inner nuclear layer.

SD-OCT analysis at 8 months duration of diabetes indicated that the overall thickness of the retina did not change in either of the genders (Fig 5A). Likewise, retinal thickness of outer nuclear layer (ONL) at 0.15, 0.30 and 0.45 mm from the optic nerve was also not significantly different in both diabetic male and female mice compared to their respective nondiabetic controls (Fig 5B). We also measured the thickness of nerve fiber layer (NFL), inner plexiform layer (IPL), inner nuclear layer (INL), as well as the total retinal thickness at 0.30 mm from the optic nerve. Fig 5C, showed that the thickness of NFL. IPL, INL or total retinal thickness were not altered in both genders.

### Visual function

**Fig 6:**
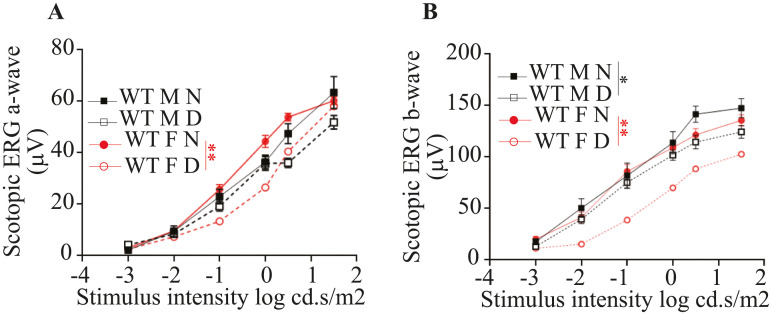
Physiologic testing on the effects of gender and diabetes on retinal function. ERG response functions were recorded to evaluate the impact of gender and diabetes on retinal function under scotopic conditions; a-wave (A) and b-wave (B). Diabetes significantly reduced b-wave in both males and females, whereas a-wave was significantly different in female mice and tend to decrease in male mice (but did not reach significance). ERG recordings of both a- and b-wave were not different between genders in N mice. Data shown as Mean ± SEM (n = 16–20 eyes). * P ≤ 0.05; ** P ≤ 0.01.

To examine overall retinal function, ERGs were recorded under dark-adapted conditions from nondiabetic and 2 months diabetic WT male and female mice. Two months duration of diabetes significantly reduced b-wave, and tended to reduce a-wave amplitude, but the results did not achieve statistical significance in male mice. Likewise, 2 months of diabetes exhibited significant reductions in a- and b-wave amplitudes in female mice (Fig 6A and 6B). Dark-adapted a- and b-wave were not different between N mice (male N versus female N mice) or D mice (male D versus female D mice) (Fig 6A and 6B).

### Leukocyte isolated from diabetic male and female mice mediated cytotoxicity to mouse retinal endothelial cells

**Fig 7:**
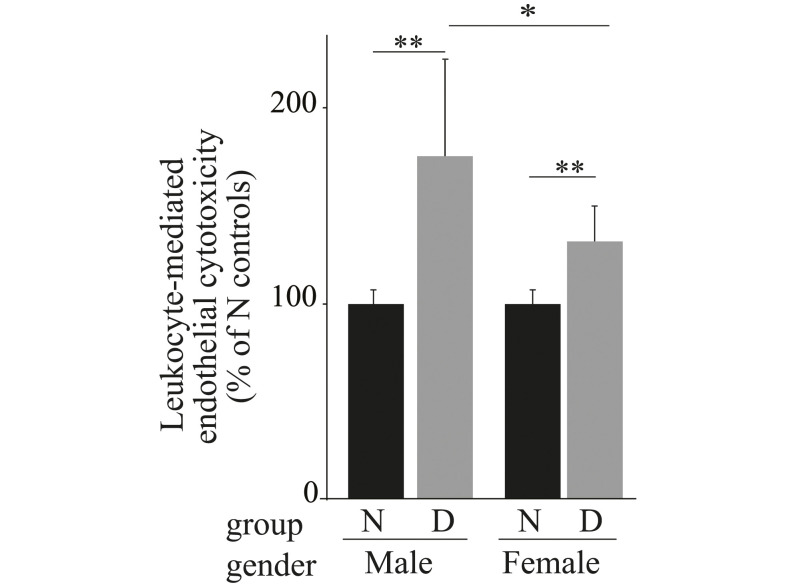
Leukocyte-mediated cytotoxicity toward retinal endothelial cells is significantly increased in both diabetic males and females compared to nondiabetic controls. Data are presented as mean ± SD, n = 6 per group. N, nondiabetic; D, diabetic. *P ≤ 0.05, **P ≤ 0.01.

Leukocytes from diabetic male mice previously have been shown to mediate cytotoxicity to retinal endothelial cells [8, 23–26], and thus has been implicated in the destruction of capillary cells in the development of DR. We investigated if leukocytes from diabetic female mice showed this same phenomenon. Fig 7 shows that leukocytes from diabetic female mice caused a significant (p<0.005) increase in endothelial cell death compared to leukocytes from nondiabetic female mice. Leukocytes from diabetic male mice showed a similar increase, but the increase using leukocytes from diabetic males was significantly greater than the increase detected using leukocytes from diabetic females.

### Eight months duration of diabetes causes a significant increase in the number of degenerated capillaries in retinas of diabetic male and female mice

**Fig 8:**
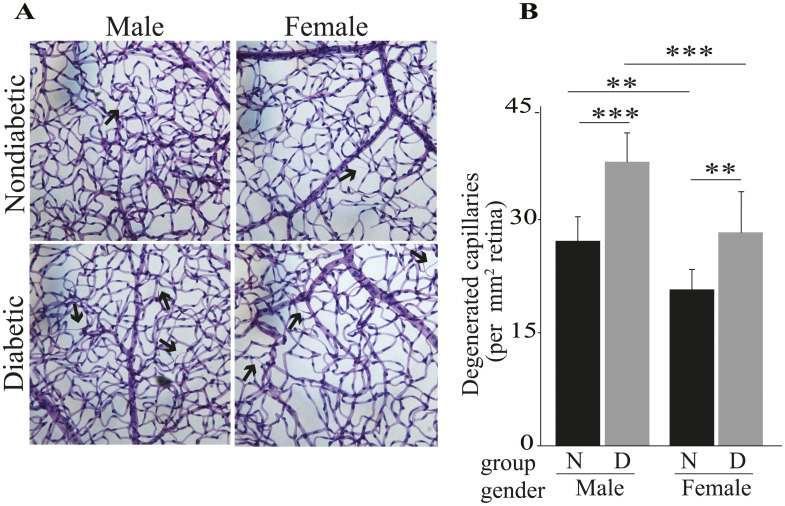
Effects of STZ-induced diabetes (8 months) on capillary degeneration in retinas from male and female mice. Representative photomicrographs depicting the retinal vasculature in nondiabetic and diabetic males and females (A). Data are graphed as degenerated capillaries per unit area of retina (B). Examples of degenerated capillaries are indicated by arrows. Data are presented as mean ± SD, n = 6 per group. N, nondiabetic; D, diabetic. **P ≤ 0.01, ***P ≤ 0.001.

As expected, duration of 8 months diabetes caused significant increase in the number of retinal capillary degeneration in male mice as compared to their nondiabetic controls. Similarly, diabetes of 8 months duration caused significant increase in the number of degenerated capillaries in females as compared to nondiabetic female controls (Fig 8A and 8B). Nondiabetic males have significantly more capillary degeneration than nondiabetic females (Fig 8B).

## Discussion

There has been a long-standing recognition that female mice are resistant to streptozotocin (STZ)-induced diabetes, therefore, the majority of studies using STZ-diabetic animals have used exclusively males [2, 4, 6]. Here, we confirm that female mice are resistant to STZ (at least, compared to males), but show that females can be made diabetic with a higher dose of the diabetogenic drug. We showed also that after diabetes has been induced, retinas from female and male mice respond similarly to the abnormal metabolic milieu. The diabetes-induced increases in retinal oxidative stress and expression of the inflammatory protein iNOS seem similar in severity between the two sexes, and the eventual diabetes-induced degenerative changes in the retinal vasculature seem comparable in severity.

It is not clear why female mice are resistant to diabetogenic effects of STZ compared to male mice. A potential mechanism has been reported is that the female hormone, 17β-estradiol (also called E2), protects pancreatic β-cells against STZ-induced oxidative stress and apoptosis [27], and the circulating E2 acts as a protective hormone, preventing β-cell apoptosis *in vivo* in both sexes [28]. Paik et al., 1982, showed that exogenous estrogens can suppress the high susceptibility of males to STZ in both BALB/c and C57BL6 mice, and conversely, that the normally STZ-resistant females become as highly susceptible to STZ as males after the administration of androgens [29]. Furthermore, male and female mice develop dramatic vulnerability to STZ-induced insulin deficiency when estradiol production is genetically suppressed [3].

In the present study, the HbA1c and glycemic response to STZ was similar in both sexes (in response to 55 mg/kg and 75 mg/kg BW in males and females respectively) at week 8 of diabetes. Our data corroborate the findings of Moore et al. [30], while other studies showed that female mice have either lower [31–33] or similar systemic response to STZ compared to male mice [34]. These differences among studies might be due to the animal strain used (C57Bl/6, CD1 or ICR mice), the number of injection of STZ given (single high dose versus lower multiple doses), or the route of STZ administration (intraperitoneal versus tail vein injection).

Diverged results have been reported with regard to total retinal thickness, inner or outer retina in diabetic rodents and patients [8, 35–40]. Martin P et al., 2004 conducted a longitudinal study for 12 weeks after the mice were declared diabetics, and measured Total retinal- ONL- and INL-thickness every 2 weeks. The authors showed that the thickness of all the measured layers were not different between diabetic and nondiabetic mice at 2, 4, 6 or 8 weeks of diabetes. The study also showed a modest, but significant decrease in the thickness of the whole retina and the inner and outer nuclear layers in mice that had been diabetic for 10 weeks [39]. Consistent with these findings, in our study we showed that total retinal thickness and the thickness of inner or outer retina layers were not statistically different between both genders that have been diabetic for 8 weeks compared to nondiabetic controls.

Oxidative stress is implicated in the development of the vascular lesions characteristic of early stage of diabetic retinopathy [17, 20, 21]. Consistent with this, in this study we demonstrated that similar to diabetic male mice, diabetic female mice showed significant increase in superoxide at 8 weeks post STZ-induced diabetes. Retinal explants isolated from male and female mice and incubated in media containing glucose concentration to mimic diabetes (high glucose) conditions or normal glucose concentrations (normal glucose) further demonstrated that both genders produced elevated amounts of superoxide when incubated with high glucose as compared to retinal explants incubated in normal glucose. The use of this *ex vivo* preparation allowed us to maintain cells in their normal cellular environment without the influence of the systemic milieu, and the results suggest that the retina of both sexes are similarly able to generate elevated levels of superoxide in high glucose.

Inflammatory changes also have been involved in the development of vascular abnormalities of diabetic retinopathy. Molecular changes that are consistent with inflammation, such as iNOS and I-CAM have been found in retinas of experimental diabetic rodents and patients in most studies [21, 22]. Consistent with the increase of iNOS in diabetic males, diabetic females also showed significant increase in the expression of iNOS after 8-weeks duration of diabetes.

It is well established that diabetes-induced retinal visual dysfunction, including b- and a-wave amplitudes, in both patients and animal models, which are recorded before severe vascular lesions appear [36, 41–43]. Our results showed that b-wave recording were significantly diminished in both male and female diabetic mice, likewise a-wave amplitudes were significantly reduced in female diabetic mice whereas in male mice diabetes tend to decrease the a-wave amplitude but the data did not achieve significance.

Leukocytes have been implicated in the pathogenesis of diabetic retinopathy [17, 21, 25]. Adherent leukocytes are temporally and spatially associated with retinal endothelial cell injury and death, and neutralization of intercellular adhesion molecule-1 and CD18 prevented leukocyte adhesion and retinal endothelial cell injury and death [44]. In the present study, we demonstrate that, similar to diabetic male mice, diabetes led to activation of circulating leukocytes in female mice, leading to significantly more killing of endothelial cells compared to nondiabetic animals.

A clinically relevant lesion that develops in early diabetic retinopathy is the degeneration of retinal capillaries, which then contributes to retinal ischemia and up regulation of growth factor (e.g. vascular endothelial growth factors). Here, we demonstrate that retinas from diabetic female mice develop capillary degeneration that is quantitatively similar to that which develops in diabetic male mice. Additionally, nondiabetic female mice showed significantly fewer degenerated capillaries compared to nondiabetic males. Degenerated retinal capillaries can be found in both nondiabetic and diabetic animals and humans, (they are more prevalent in diabetes), therefore the significance of the observed difference between degenerated capillaries in nondiabetic male and female mice is unclear and warrants additional investigations.

In summary, we demonstrate that diabetes can be induced in female C57BL/6J mice using STZ, however they do require higher dose than do male mice. Diabetic female mice develop molecular changes (superoxide generation and the induction of the inflammatory protein iNOS), as well as histopathologic abnormalities (retinal capillary degeneration) similar to those that develop in diabetic male mice. The use of both male and female mice not only is crucial to study the pathogenesis of diabetic retinopathy, but it is also important for the identification of the therapeutic agents that are safe and have high efficacy for both sexes.
